# Learning Curve and Initial Outcomes of Full-Endoscopic Posterior Lumbar Interbody Fusion

**DOI:** 10.3389/fsurg.2022.890689

**Published:** 2022-04-28

**Authors:** Renchun Tan, Xin Lv, Pengfei Wu, Yawei Li, Yuliang Dai, Bin Jiang, Bolin Ren, Guohua Lv, Bing Wang

**Affiliations:** ^1^ Department of Spine Surgery, The Second Xiangya Hospital, Central South University, Changsha, Hunan, China; ^2^ Department of Orthopedic Surgery, The Second Xiangya Hospital, Central South University, Changsha, Hunan, China; ^3^ Hunan Key Laboratory of Tumor Models and Individualized Medicine, The Second Xiangya Hospital, Central South University, Changsha, China

**Keywords:** full-endoscopic, posterior approach, interbody fusion, minimally invasive surgery, learning curve

## Abstract

**Study Design:**

This was a retrospective cohort study.

**Objective:**

We evaluated the feasibility, safety, and accuracy of full-endoscopic posterior lumbar interbody fusion (FE-PLIF) by assessing the learning curve and initial clinical outcomes.

**Summary of Background Data:**

Low back pain is one of the crucial medical conditions worldwide. FE-PLIF has been reported to be a minimally invasive method to treat mechanical low back pain, but there lacks a thorough evaluation on this new technique.

**Methods:**

The patients were divided into three groups in the order of operating date, implying that Group A consisted of the initial 12 cases, Group B the subsequent 12 cases, and Group C the last 12 cases. The data of patients were reviewed for gender, age, preoperative symptoms, satisfaction, as well as clinical outcomes demonstrated by visual analog scale (VAS). The operative time and intraoperative fluoroscopy were recorded to demonstrate the learning curve and the extent of radiographic exposure. Statistical significance was set at a *p* < 0.05 (two-sided).

**Results:**

The patients enrolled in this study were followed up at an average of 1.41 ± 0.24 years. Overall, patients were satisfied with the surgery. The average number of intraoperative fluoroscopy was 6.97 ± 0.74. A significant improvement was observed in the VAS of both lumbar pain and leg pain. The overall fusion rate was 77.7%. Complications were reported in two patients in Group A, one in Group B, and none in Group C. The average operative time showed a trend of gradual decline. The learning curve was characterized using a cubic regression analysis as *y* = –27.07x + 1.42x^2^–0.24x^3^ + 521.84 (*R*^2^ = 0.617, *p* = 0.000).

**Conclusions:**

FE-PLIF is an effective and safe method for treating low back pain caused by short-segmental degenerative diseases. The learning curve of this technique is steep at the initial stage but acceptable and shows great potential for improvement.

## Introduction

Low back pain is one of the crucial medical problems worldwide, especially in low- and middle-income countries that lack enough resources to treat it (1). Mechanical lower back pain intrinsically arises from changes in human body structures such as the spine, intervertebral disc, and the surrounding soft tissue (2). In the case of ineffective conservative treatment, the mainstay treatment of mechanical lower back pain caused by degenerative lumbar diseases involves lumbar discectomy together with interbody fusion. Posterior lumbar interbody fusion (PLIF) has been widely used for significantly reducing the pain, restoring sagittal profile, decreasing complications, as well as gaining good fusion rates and long-term stability (3–5). However, conventional open posterior surgery is associated with the risk of nerve root injury and dural tear, as well as longer operation time, more blood loss, and extensive scar formation within the spinal canal (6). Therefore, several new techniques have been developed to achieve better clinical outcomes, easier process and less trauma.

The past few years have witnessed a trend toward minimally invasive, accurate, and intelligent procedures for the surgical therapy of low back pain caused by degenerative lumbar diseases**.** As a minimally invasive surgery, the newly reported technique of full-endoscopic posterior lumbar interbody fusion (FE-PLIF) has been recognized as a safe and reliable method for its clear visualization and minimal damage as it uses a rigid rod-shaped endoscope, which integrates the working channel together with lighting, camera and irrigation system (7). In addition, good decompression and accurate intervertebral cage insertion are obtained with the assistance of the endoscope. The percutaneous pedicle screw implantation provides local stability similar to that endowed by traditional procedures. However, full-endoscopic surgery requires effective hand-eye cooperation and identification of under-endoscopic anatomic structures that may lead to a steep learning curve, limiting its applications (8). In this study, we systematically evaluated the learning curve of FE-PLIF and reported the initial clinical outcomes together with our preliminary experience to further provide a thorough assessment of the safety, accuracy, and feasibility of FE-PLIF.

## Materials and Methods

### Patients

The study retrospectively enrolled the first 36 patients who underwent FE-PLIF surgery consecutively in our hospital. The diagnosis was confirmed by two clinical professors based on a combination of clinical symptoms and imaging evidence including X-ray, computed tomography (CT), and magnetic resonance imaging (MRI). The patients were divided into three groups in the order of operation date, implying that Group A consisted of the initial 12 cases, Group B the subsequent 12 cases, and Group C the last 12 cases. The operations were performed by two fellowship-trained spine surgeons. The clinical demographic features and radiographic features of the patients are shown in **Tables 1, 2**.

**Table 1:** Clinical demographic data of three groups that underwent FE-PLIF.

Parameters	Group A	Group B	Group C	*p* value
Sex ratio (female/male)	6/6	6/6	7/5	0.895
Age (years, mean ± SD)	50.92 ± 11.16*	49.42 ± 9.08	50.00 ± 7.26	0.528
Duration of symptoms (days, mean ± SD)	78.83 ± 34.92	75.00 ± 49.41	92.50 ± 42.88	0.580

**Table 2:** Clinical radiographic data of three groups that underwent FE-PLIF.

Variable	Group A	Group B	Group C
L4/5 disc herniation	6	5	4
L5/S1 disc herniation	2	5	5
L4/5 spinal canal stenosis	3	1	1
L4/5 spondylolisthesis	–	–	1
L5/S1 spondylolisthesis	1	1	1

The inclusion criteria were (1) discogenic low back pain, (2) single or double segmental degenerative lumbar stenosis mainly caused by lumbar disc herniation, facet joint hyperplasia, or hypertrophy of the ligamentum flavum, (3) degenerative lumbar spondylolisthesis up to Grade I according to the Meyerding standard, and (4) other single or double segmental degenerative lumbar diseases requiring stability and fusion. The exclusion criteria of this study were (1) the presence of a significant spinal deformity, (2) developmental or multi-segmental (over two) lumbar spinal stenosis, (3) severe spondylolisthesis hard to restore without an open procedure, (4) unclear location of the responsible segment or imaging data inconsistent with the patient’s symptoms, and (5) intolerance to FE-PLIF for other reasons such as severe cardiopulmonary disease and previous lumbar surgery.

### Surgical Technique

**Figure 1:**
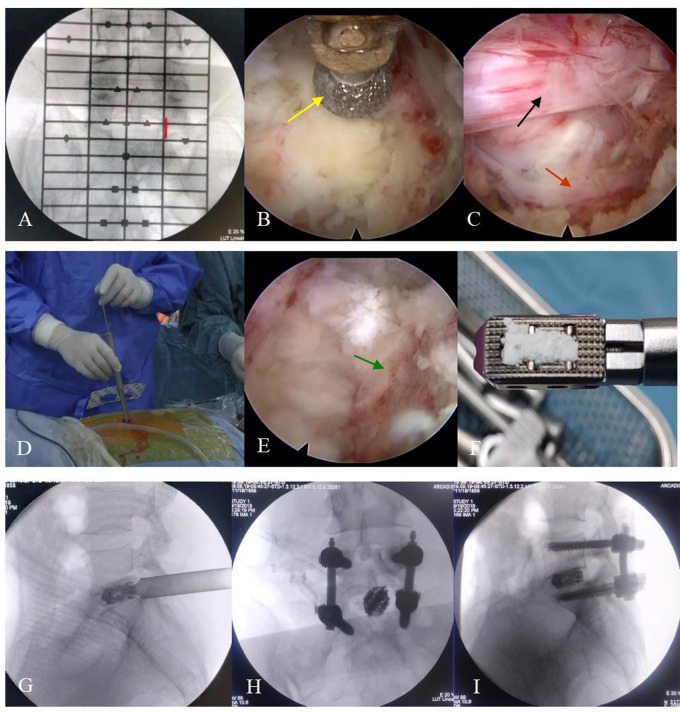
The surgical steps. (**A**) The positioning device is used to identify the responsible segment and decide the location of the incision (red line). (**B**) The articular process (yellow arrow) is polished by endoscopic burrs. (**C**) The vessel (red arrow) and nerve root (black arrow) are exposed before entering into the spinal canal. (**D**) The working channel is replaced before placing the expandable cage. (**E**) The cartilaginous endplate is removed and the bony endplate (green arrow) is exposed. (**F**) The cage is grafted with the bone before implantation. (**G–I**) The position of the cage and percutaneous pedicle screws is confirmed by “C-arm” fluoroscopy when placed.

All patients enrolled in our study were operated on using the iLESSYS Delta Endoscopic System (Joimax GmbH, Karlsruhe, Germany) for visualization. Under general anesthesia and neuromonitoring, the patient was placed in the prone position on a radiolucent table with the abdominal suspension to reduce abdominal pressure and therefore decrease bleeding. Routine disinfection, sterile towel sheet spread, and incision protective film affixation were performed. After confirming the surgical position and angle by “C-arm” (**Figure 1A**), a longitudinal incision of about 1.2 cm in length was made above the responsible segment with a No. 11 blade. Serial dilators were advanced step-by-step until palpating the lamina. A working cannula with an outer diameter of 13.7 mm and an inner diameter of 10.2 mm was inserted through the dilator. Next, the dilaters were removed, and the “C-arm” fluoroscopy was performed to confirm the location of the cannula.

Afterward, an endoscopic system with an irrigating channel, which had an outer diameter of 10 mm, an inner diameter of 6 mm and a view angle of 15-degree, was connected and placed into the working cannula. The ipsilateral laminectomy, removal of the ligamentum flavum and medial facetectomy were performed by endoscopic burrs, Kerrison punches and osteotomes (**Figure 1B**), and the nerve root inside the spinal canal was exposed layer by layer during this process (**Figure 1C**). With the nerve root carefully protected, a standard full-endoscopic discectomy was performed using endoscopic forceps.

The working channel was then replaced by a dedicated fork-shaped cannula with an inner diameter of 11.5 mm (**Figure 1D**) for cage implantation. The intervertebral space was further treated with serial reamers, curettes, and rasps until the cartilage endplate was completely peeled off and the bony endplate was entirely exposed for better fusion. Harvested local bones from laminectomy and arthrotomy performed earlier were inserted into the anterior disc space, and an expandable titanium cage (Shanghai Reach Medical Instrument Co., Ltd., Shanghai, China) was placed into the intervertebral space along the working channel. Its location was confirmed by “C-arm” fluoroscopy. The cage location, nerve root relaxation, residual nucleus tissue, and active bleeding inside and outside the spinal canal were rechecked under the endoscopic view. Next, the endoscope and working channel were withdrawn. Bilateral percutaneous pedicle screws and connecting rods with appropriate length were placed on the upper and lower vertebral bodies and fixed (Zina; Sanyou, Shanghai, China). If the patient was diagnosed with lumbar spondylolisthesis, the reduction was required simultaneously. The “C-arm” fluoroscopy was performed again to confirm the position of the internal fixation device. Next, the incision was sutured layer by layer. No drainage was required.

### Outcome Measures

We used VAS which rages from 0 to 10 to evaluate the pre- and post-operative clinical results. Operative time, blood loss, times of intraoperative X-ray fluoroscopy, length of hospital stay, complications, and rate of conversion to an open procedure were recorded. The satisfaction of patients was scored using the Macnab criteria. The fusion rates were evaluated by using Bridwell’s fusion grading system on computer tomography scans or radiographs at the last follow-up. All radiographs in this study were assessed by two independent researchers. Through the discussion with another independent expert, different opinions on fusion healing were reconciled and reached a consensus.

### Statistical Analysis

SPSS version 22.0 (SPSS 109 Inc., Chicago, IL) was used to conduct statistical analysis. The data were analyzed using independent sample *t*-test, paired *t*-test, chi-square test, one-way analysis of variance, and regression analysis. Statistical significance was set at a *p* < 0.05 (two-sided).

## Results

### Clinical Outcomes

**Figure 2:**
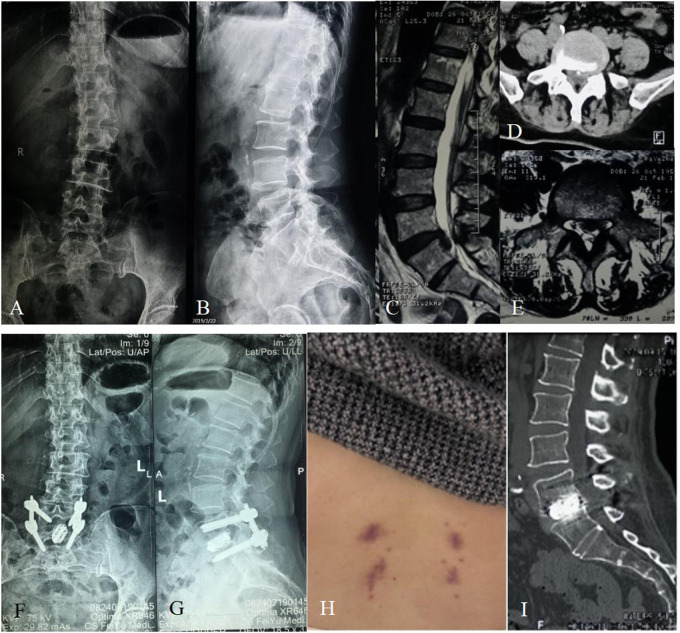
Sixty-two-year-old female with over 10-year lumbar pain and 1-week leg pain. (**A**,**B**) Preoperative X-ray showing the patient had a slightly degenerative lumbar scoliosis and had no lumbar spondylolisthesis or apparent lumbar instability. (**C**) Preoperative sagittal MRI showing the patient had an L5/S1 disc herniation with downward prolapse. (**D**,**E**) Preoperative cross-sectional CT and MRI showing that the herniated disc with calcification oppressed the left nerve root of S1. (**F**,**G**) The postoperative X-ray showing that the cage and pedicle screws were complete and in position. (**H**) There was a very small incision scar that healed well at the follow-up of 3 months after surgery. (**I**) The postoperative CT showing a sign of probable fusion within the interbody of L5/S1 at the follow-up of 6 months after surgery.

**Figure 3:**
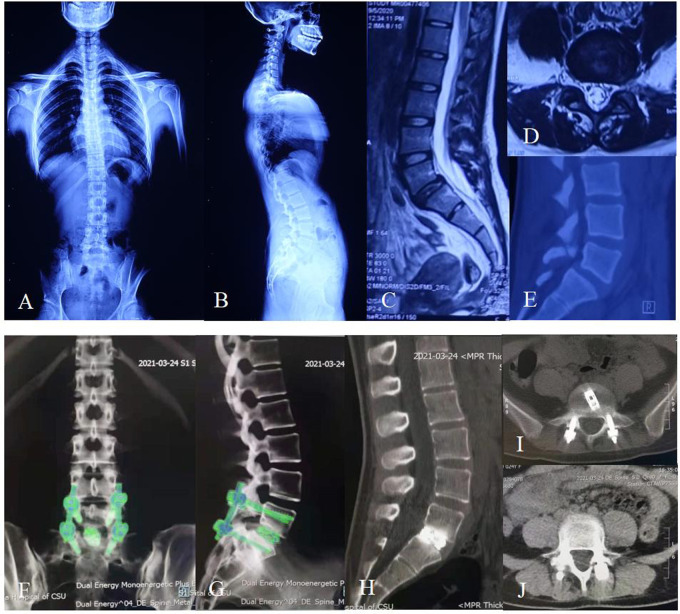
Twenty-four-year-old female with 10-month lumbar pain and half-month left leg pain. (**A**,**B**) Preoperative X-ray showing the patient had a degenerative L5/S1 spondylolisthesis up to Grade I according to the Meyerding standard and had a slightly degenerative lumbar scoliosis. (**C**) Preoperative sagittal MRI showing the patient had an L5/S1 spondylolisthesis and multi-level disc degeneration. (**D**) Preoperative cross-sectional MRI showing that the spondylolisthesis in L5/S1 led to a stenosis of left lateral recess. (**E**) Preoperative sagittal CT showing an isthmus fissure in L5 and spondylolisthesis in L5/S1. (**F**,**G**) The postoperative dual energy CT showing that the cage and pedicle screws were complete and in position at the follow-up of 6 months after surgery. (**H–J**) The postoperative CT showing a sign of fusion within the interbody of L5/S1 at the follow-up of 9 months after surgery, and the fixation instruments were complete and in position.

All of the 36 patients enrolled in this study had undergone FE-PLIF between 2019 and 2021. The radiological measurement data revealed 27 cases of lumbar disc herniation, 5 cases of lumbar spinal canal stenosis, and 4 cases of lumbar spondylolisthesis. All patients underwent FE-PLIF successfully without conversion to open surgery. The blood loss was less than 70 mL in all patients; one patient required postoperative drainage in Group A due to intraoperative dural tear. The average number of intraoperative fluoroscopy performed was 6.97 ± 0.74. The patients enrolled in this study were followed up at an average of 1.41 ± 0.24 years (range: 1–2 years). The majority of patients had immediate relief in pain and dysesthesia. The incidence of complications was 8.3%. One case of dural tear and one case of incomplete reduction requiring open-access revision after 3-month follow-up in group A, one case of postoperative nerve root symptom in group B, and no complications in group C were reported. Patient satisfaction measured using the Macnab criteria showed the surgical outcomes were excellent in 27 (75%) patients, good in 8 (22.2%) patients, and fair in 1 (2.7%) patient with no poor assessment. There was no recurrence of clinical symptoms until the final follow-up (**Figures 2** and **3**).

**Figure 4:**
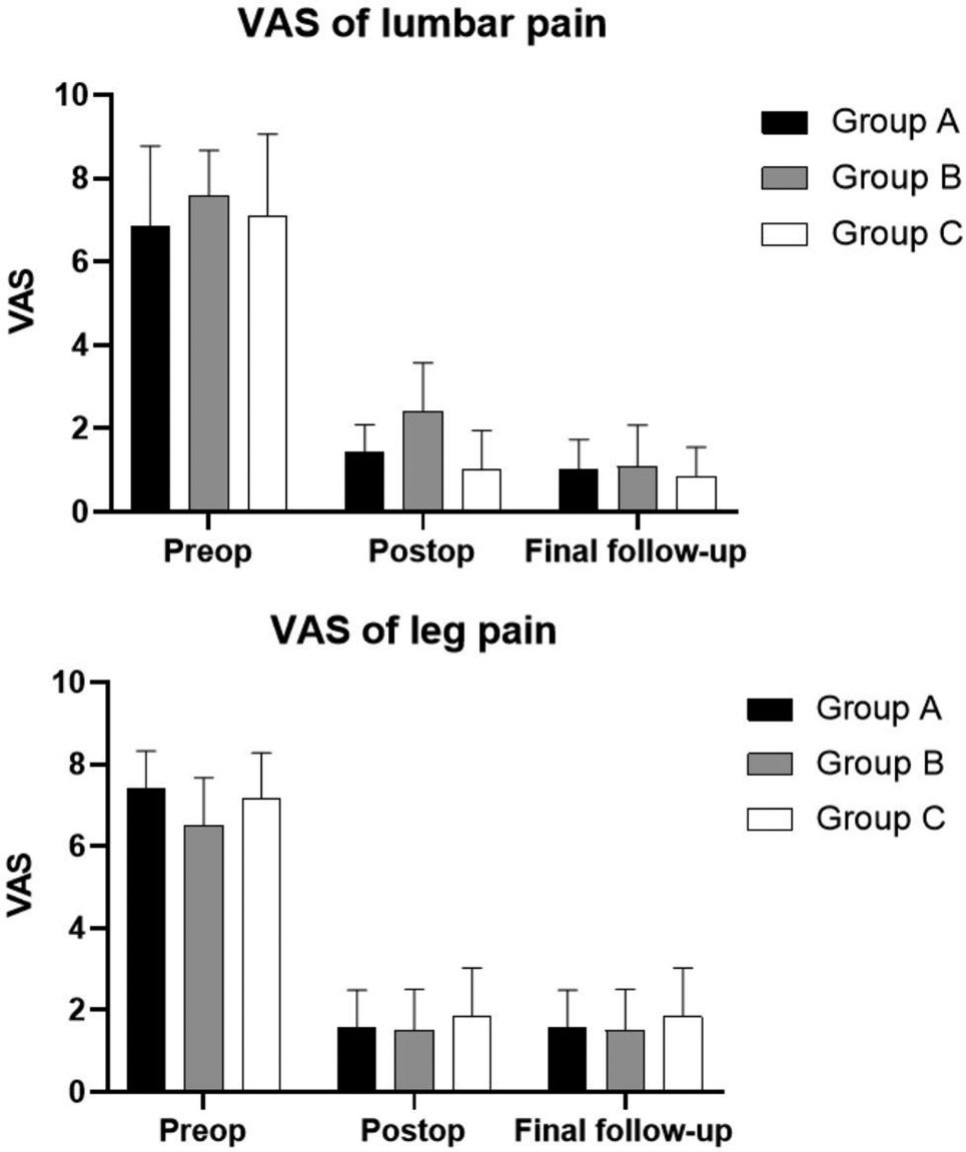
Mean values and standard errors of visual analog scores (VAS) in three groups. (**A**) VAS of lumbar pain in three groups. (**B**) VAS of leg pain in three groups. Abbreviations: Preop, preoperative; postop, postoperative; VAS, visual analog score.

The quantified clinical outcomes are shown in **Table 3**, **Figure 4A**,**B**. In Group A, the average postoperative hospitalization stay was 5.62 ± 1.69 days (range: 3–9 days). Case 1 stayed in the hospital after surgery for an especially longer duration than other patients because the complete observation was needed for the first case to ensure safety. The hospitalization duration of Case 5 was extended for the removal of the drainage tube and the recovery of dural tear. A significant improvement in the VAS of lumbar pain at day 1 after the surgery was recorded compared with preoperative VAS (*p* < 0.001). However, no statistical difference was found between day 1 after the surgery and the final follow-up (*p* = 0.137). As for the VAS of leg pain, the outcome at the final follow-up was significantly improved compared with that at day 1 after the surgery (*p*-value between preoperative and postoperative VAS was less than 0.001, and was 0.009 between postoperative and final follow-up VAS). In Group B, the average hospitalization stay was 5.50 ± 2.07 days (range: 3–8 days). The VAS of lumbar pain and leg pain were both significantly improved at day 1 after the surgery compared with the preoperative VAS (*p* < 0.001, both) and improved tremendously at the final follow-up compared with day 1 after the surgery (*p* = 0.008 and 0.026, respectively). Case 20 stayed in the hospital for a long time because she felt pain in the right leg that increased while walking. The pain did not subside immediately after the surgery and turned worse, which was believed to be related to the intraoperative traction for the nerve root. After hormone and dehydration therapy to relieve neuro edema, the pain reduced slightly 7 days after the surgery. In Group C, the average hospitalization stay was 4.38 ± 2.07 days (range: 2–9 days). There was a significant improvement on day 1 after the surgery in both VAS of lumbar and leg pain (*p *< 0.001, both). Statistical difference was only found in VAS of leg pain between day 1 after the surgery and the final follow-up (*p* = 0.003), but there was no significant difference in those of lumbar pain (*p* = 0.615). As shown in **Figure 4**, no statistical difference was found in the VAS of leg pain among all the three groups in each period. Furthermore, no statistical difference was found among them in the VAS of preoperative and final follow-up lumbar pain. The VAS of postoperative lumbar pain showed a different status; the VAS of postoperative lumbar pain in Group B was slightly higher than that of Groups A (*p* = 0.017) and C (*p* = 0.004).

**Table 3:** Clinical outcomes of three groups that underwent FE-PLIF.

Parameters		Variable			*p* value
		Group A	Group B	Group C	
VAS of lumbar pain	Preoperative	6.83 ± 1.95	7.58 ± 1.08	7.08 ± 1.98	0.559
	Postoperative	1.42 ± 0.67	2.41 ± 1.16	1.00 ± 0.95	0.003*
	Final follow-up	1.00 ± 0.74	1.08 ± 1.00	0.83 ± 0.72	0.754
VAS of leg pain	Preoperative	7.42 ± 0.90	6.75 ± 1.36	7.17 ± 1.11	0.362
	Postoperative	1.58 ± 0.90	1.50 ± 1.00	1.83 ± 1.19	0.718
	Final follow-up	0.67 ± 0.65	0.50 ± 0.67	0.58 ± 0.67	0.829
Postoperative hospitalization duration (days)		5.62 ± 1.69	5.50 ± 2.07	4.38 ± 2.07	0.385
Cases of complications		2	1	0	–
Fusion rate (%)		75	91.6	66.6	0.345
Definite fusion		9	11	8	–
Probable fusion		2	1	0	–
Non-union		1	0	4	–

*VAS, visual analogue score.*

* *p < 0.05, the difference between groups was statistically significant.*

According to Bridwell’s fusion grading system, there were 9 cases of definite fusion and 2 case of probable fusion in group A, 11 cases of definite fusion and 1 case of probable fusion in group B, and 8 cases of definite fusion in group C. At 1-year follow-up, the overall fusion rate with definite grade reached 77.7%. The fusion rate with definite grade reached 75% in group A, 91.6% in group B and 66.6% in group C (**Table 3**). No significant difference was observed in fusion rate among three groups at 1-year follow-up (*p* = 0.345).

### Results for the Learning Curve

**Figure 5:**
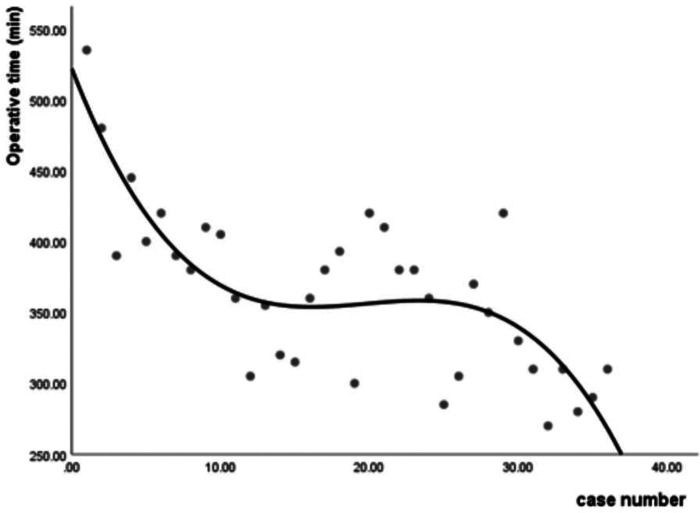
The learning curve of FE-PLIF.

The operative time was recorded to evaluate the learning curve of FE-PLIF. The average operative time was 410.00 ± 58.13 min in Group A (range: 305–535 min), 364.42 ± 37.42 min in Group B (range: 300–420 min), and 319.17 ± 42.90 min in Group C (range: 270–420 min). A statistical difference was found among the three groups (*p *< 0.001). Further analysis found that statistically significant differences existed not only between Group A and Group B (*p* = 0.032), but also between Group B and Group C (*p* = 0.012). The median of operative time appeared in Case 11. Case 1 in Group A experienced the longest operative time among all patients in that group. In Group B, Case 20 experienced a longer operative time than other patients because her herniated disc was extremely large, making it difficult to perform complete decompression. In Group C, Case 29 experienced the longest operative time in this group because the spondylolisthesis had lasted a few years and thus was hard to reduce. No statistical difference was present among the operative time of different diseases (*p* = 0.337). The learning curve shown in **Figure 5** was characterized using a cubic regression analysis (*y* = –27.07x + 1.42x^2^–0.24x^3^ + 521.84, *R*^2^ = 0.617, *p* = 0.000).

## Discussion

Methods to achieve better clinical outcomes and fewer complications have always drawn the attention of surgeons since spinal fusion was first described in 1911(9). Although several surgical approaches have been developed, the best choice remains controversial, especially after the introduction of the endoscopic lumbar discectomy technique in 1988 that brought surgical spine into the minimally invasive era (10–15). Both posterior interlaminar and lateral transforaminal approaches are frequently used procedures. Previous studies have reported similar efficacy of these two established techniques (16). Compared with PLIF, the transforaminal approach was invented to reduce the chance of damaging the nerve root, which was achieved by unilateral exposure during decompression, unilateral cage interference during insertion, as well as less traction due to lateral approach (17). However, this technique requires expanded foraminal and thus has its limitations, such as the trauma caused by resection of ventral articular process, potential risk of injuring exiting nerve root, and difficulty in access caused by the high iliac spine (18–20). The posterior approach is superior in bilateral decompression, familiar overlooking angle of view, good visualization, as well as the ability to deal with several types of herniations such as huge herniation and herniation with calcification (21–23). However, this technique suffered from high invasiveness. The FE-PLIF used in our study provides a modified method. The application of a working channel with an inner diameter of over 10 mm ensured a rapid and convenient decompression due to a large operative space. Moreover, the interlaminar approach reduced the damage of the articular process by arthroplasty as well as avoided the exit of the nerve root necessary for the transforaminal approach (24). In addition, the large fork-shaped cannula enabled the use of an expandable cage, which benefitted in restoring disc height and rebuilding lumbar lordosis (25). A combination with percutaneous pedicle screw, which has been widely applied and studied in spine trauma (26–28), helped in achieving stability similar to that of open instrumentation. Overall, the technique of FE-PLIF realized excellent decompression using endoscopy and a large working channel, achieved outstanding stabilization through an expandable cage and pedicle screw fixation in a minimally invasive manner. In this way, FE-PLIF has the strength of wide application in treating lower lumbar vertebrae with symptomatic bilateral recess stenosis, high iliac crest, large L5 transverse process, large articular process, narrow intervertebral disc space and spondylolisthesis lower than grade II (29). In our study, the VAS of lumbar pain and leg pain significantly decreased after surgery in all groups, which affirmed the curative effect of FE-PLIF. Partial patients in showed a better improvement in VAS at the follow-up compared with postoperative VAS. Previous studies showed that different ways of exercising, habits, and physical therapy after the surgery could differently benefit recovery (30, 31). Therefore, more detailed studies are required to identify the reason for this difference. The VAS of postoperative lumbar pain in Groups A and C was significantly better than that of Group B. Considering there was no statistically significant difference between the preoperative lumbar pain, the different postoperative effects could be ascribed to increased skilled operations and nursing with time. Complications including nerve root symptoms resulting from excessive intraoperative traction, incomplete decompression requiring open-access revision, and dural tear were reported in Groups A and B. In our study, patients underwent FE-PLIF reached a difinite fusion of 77.7% at 1-year follow-up, which was similar to previous study (32). The complications showed a decreasing trend with increased experience, suggesting that FE-PLIF is generally an effective, safe, and reliable method for decompression and stabilization. However, the endoscopic and percutaneous procedures require repeated fluoroscopy during operation to confirm the position of instruments such as the working channel, cage, and pedicle screws, which may increase radiographic exposure of both surgeons and patients. Radiation exposure is known to harm the human body, especially in early life (33, 34). As per our experience, minimally invasive spinal surgery is more popular among young patients. Therefore, the control of radiation exposure should be seriously considered. The indication of FE-PLIF should be strictly controlled, and various techniques including navigations could be combined with FE-PLIF to reduce fluoroscopy frequency (35–37). Certain other improvements could also be made. The expandable cage used in our study was made of titanium, which may probably bring out the problems including settlement related to metal particles, controversial fusion rates, and the need for a large amount of bone graft. Several new materials have been developed to reduce such shortcomings such as polyether ether ketone (38). In addition, similar to other surgical techniques with pedicle screw implantation, when the patient suffers from osteoporosis, strengthening methods such as a screw with bone cement should be considered (39, 40).

To the best of our knowledge, this is the first study focusing on the learning curve of FE-PLIF. Our study suggested that the learning curve of FE-PLIF was steep at the initial stage. The overall operative time of FE-PLIF appeared a little long, which may increase the risk of hidden blood loss and anesthetic accident—play an important role in perioperative rehabilitation (41, 42). The reason may be related to repeated fluoroscopy, lack of experience, as well as the addition of cage and percutaneous screw insertion. The significant difference in the operative time among groups showed there was a trend of gradual decline along with the increasing number of operations. The learning curve shown in **Figure 5** suggested that after the initial ten-time practices, the skills could be well mastered, and the downward trend of the learning curve shows a great potential to complete the operations in a shorter time before reaching a stable performance. It could be inferred that the operative time may be controlled within 3 h with increased experience. To increase safety and efficiency, more advanced training in endoscopic procedures for surgeons is advocated. Furthermore, the development of endoscopic instruments could benefit the improvement in the learning curve of FE-PLIF.

An unneglected limitation of this study is the limited number of cases enrolled. Compared with the outcome reported by Kim et al, in which the technique of bi-portal endoscopy-assisted lumbar interbody fusion required approximately 34 cases to reach an adequate performance level (43), the limited number of cases may increase the statistical error and decrease the accuracy of the evaluation of the learning curve in our study. Considering the difficulty for beginners to adapt to both endoscopic lumbar operation and percutaneous pedicle screw implantation simultaneously, more cases and surgeons should be enrolled into studies for a better evaluation. In addition, our study was based on a short-term follow-up, which is not as reliable as long-term clinical outcomes, given that certain complications, such as mechanical complications for internal fixation, chronic low back pain, and failed fusion can appear at a long time after the surgery. Further studies are expected to include larger samples, report outcomes with longer terms, and use more indicators to better evaluate the safety and efficacy of FE-PLIF.

## Conclusion

According to our results, FE-PLIF is a safe and effective method to treat low back pain caused by short-segment degenerative diseases. The learning curve was initially steep, turned stable after 10 times of practice and showed great potential in shortening the operation time into lower than 3 h.

## Data Availability

This was a retrospective study based on the true follow-up materials of cases undergone surgeries. All the data for patients' relevant information were available in the system of our hospital.
